# Supplementary material for: Simulated Disperser Analysis: determining the number of loci required to genetically identify dispersers
Source: PeerJ. 2018 Mar 29;6:e4573. doi: 10.7717/peerj.4573 (PMC5878929; doi:10.7717/peerj.4573)
Supplement: Table S1 — Characterisation of microsatellite loci in Sturnus vulgaris (N = 94) including locus name, GenBank accession number, primer sequences, repeat motif, number of alleles and allele size range. [file peerj-06-4573-s001.docx]

Supplementary table 1: Characterisation of microsatellite loci in *Sturnus vulgaris* (*N*=94) including locus name, GenBank accession number, primer sequences, repeat motif, number of alleles and allele size range.

| **Locus**  **GenBank** | **Primer Sequence (5’ to 3’)*** | **Repeat Motif** | **Number**  **of Alleles** | **Size Range*** | **Dye** | **Multi-plex** |
| --- | --- | --- | --- | --- | --- | --- |
| *Svu002^*  KF535925 | F: TACCAACCAGCCACAGATTG  R: TACCAGCTGCATTTCCTCCT | GT | 12 | 100-129 | NED | 1 |
| *Svu004*  KF535926 | F: ATTCCACTACCAGCCACCAG  R: AGTGCAGGTGTTGGAAAGGT | CATC | 11 | 220-255 | VIC | 1 |
| *Svu005*  KF535927 | F: TTTCGCAAGCACAATTTCTTT  R: ACTGGCTCTCCTGAGTCCAA | CCAT | 15 | 150-224 | 6-FAM | 1 |
| *Svu006*  KF535928 | F: CCTTTCCAATTCTCTTCCCC  R: AGTCAGCCACCAGGTACCAC | GTGA | 14 | 77-144 | VIC | 1 |
| *Svu007*  KF535929 | F: CCAAGGGATAGCAATAGGCA  R: CTGGCCCAGTTAGCATAGAGA | TAGG | 12 | 153-184 | PET | 1 |
| *Svu010^*  KF535930 | F: CAGTTCCTGGGTTGGTTTTG  R: ATTTCATGCTTTGTTGTGCG | AC | 9 | 98-130 | NED | 2 |
| *Svu012*  KF535931 | F: CCTCATCCTCCTAGCACAGC  R: AGGGAGAAGCTGGTGTCAGA | CA | 9 | 128-171 | PET | 2 |
| *Svu013*  KF535932 | F: TGTACTGGCTAACGGAGCAC  R: GCTGTGATTGTGAGTCGTGG | GT | 13 | 86-115 | 6-FAM | 2 |
| *Svu015*  KF535933 | F: CAGCACAACAGAGCAGGAGA  R: AATTTGCTCCCCACAGAAGA | AAAC | 5 | 123-139 | 6-FAM | 3 |
| *Svu016*  KF535934 | F: GCTTCTATTATCTTCTGCCATCCT  R: GATGAGGCATGGATGGAGTG | TCCA | 9 | 139-169 | PET | 3 |
| *Svu017*  KF535935 | F: TTGAACAAGCTGGTCTTGGA  R: ATATCCACGGAACAGAGCCA | GATA | 12 | 244-295 | 6-FAM | 3 |
| *Svu018*  KF535936 | F: TGAAAGGCTTTTGTAGCAGTCA  R: ACTGGATAAGCTTCCTGGGG | TAGA | 11 | 168-215 | 6-FAM | 2 |
| *Svu021*  KF535937 | F: AAGGCTGACGTGGCAATTAT  R: CACGTGAGGTGTGCTTGTCT | AC | 5 | 89-100 | NED | 3 |
| *Svu022*  KF535938 | F: GCGCCTGTGTTTTGCATTAT  R: TCAGGTACTGCTTGGTGCAT | CA | 12 | 92-121 | VIC | 3 |
| *Svu025*  KF535939 | F: AGCTCACTGGAGCAGCCTAC  R: TCACAGCATTCAGGGAATCA | GT | 8 | 104-123 | PET | 3 |
| *Svu030*  KF535940 | F: ACACACAAGAGGGAACCTGG  R: TTTGGTGGATGGATAGAGGG | TA | 6 | 186-214 | VIC | 2 |
| *Svu033*  KF535941 | F: TGACATGATCACAGTGGAAGG  R: TGAGTCACTGCATAGGCATACA | ATCA | 8 | 74-109 | 6-FAM | 1 |
| *Svu034*  KF535942 | F: ACTCTGGCATAGGAGGGGAC  R: GCTCAGCATGGCTGTATTGA | CA | 13 | 163-200 | VIC | 3 |
| *Svu038*  KF535943 | F: CCTGTAGGACCATGGAGCAT  R: TAGACCGGAACCTTGTCTGG | ATGG | 7 | 242-266 | NED | 1 |
| *Svu039*  KF535944 | F: CCACTGCTTCTCTGGGGTAA  R: GCATAGCTACACCGTGGGAT | ATCA | 4 | 93-112 | PET | 1 |

* Universal primer sequence removed. ^These loci were not in Hardy-Weinberg equilibrium and were removed from downstream analyse

# Simulated Disperser Analysis: determining the number of loci required to genetically identify dispersers

Adam P.A. Cardilini^1^, Craig D.H. Sherman^2^, William B. Sherwin^3^, Lee A. Rollins^2,3^

^1^ Faculty of Science, Engineering and Built Environment, Deakin University, 221 Burwood Hwy, Burwood, Victoria 3125, Australia

^2^ Centre for Integrative Ecology, School of Life and Environmental Science, Deakin University, 75 Pigdons Rd, Waurn Ponds, Victoria 3216, Australia

^3^ Evolution & Ecology Research Centre, School of Biological, Earth and Environmental Sciences, The University of New South Wales, Sydney NSW 2052, Australia

Keywords: Ecological Genetics, Population Genetics, Power Analysis, Migrant, GeneClass2

Corresponding Author:

Adam P.A. Cardilini

Faculty of Science, Engineering and Built Environment, Deakin University, 221 Burwood Hwy, Burwood, Victoria 3125, Australia

Email: a.cardilini@gmail.com
