# Supplementary material for: Simulated Disperser Analysis: determining the number of loci required to genetically identify dispersers
Source: PeerJ. 2018 Mar 29;6:e4573. doi: 10.7717/peerj.4573 (PMC5878929; doi:10.7717/peerj.4573)
Supplement: Table S2 [file peerj-06-4573-s002.docx]

***Supplementary table 2.*** Papers included in the literature search. The number of loci and the *F_ST_*_­_ value (global or average pairwise *F_ST_*_­_) are given.

| References | Common *-*me | Species *-*me | No. of Loci | Fst |
| --- | --- | --- | --- | --- |
| Rugman-Jones *et al.* 2007 | *-* | *Scirtothrips perseae* | 4 | 0.115 |
| Bo*-*nomi *et al.* 2016 | Atlantic cod | *Gadus morhua* | 4 | *-* |
| Scott *et al.* 2005 | *-* | *Helicoverpa armigera* | 5 | *-* |
| Chevolot *et al.* 2006 | thornback ray | *Raja clavata* | 5 | 0.013 |
| Wilson *et al.* 2007 | Walleye | *Sander vitreus* | 5 | *-* |
| Miko *et al.* 2011 | St. Francis satyr butterfly | *Neonympha mitchellii francisci* | 5 | 0.078 |
| Robinet *et al.* 2011 | pine processio*-*ry moth | *Thaumetopoea pityocampa* | 5 | *-* |
| Graciš *et al.* 2012 | spur-thighed tortoise | *Testudo graeca* | 5 | 0.14 |
| Montoya-Maya *et al.* 2016 | Coral | *Platygyra daedalea* | 5 | 0.105 |
| Kraaijeveld-Smit *et al.* 2005 | Mallorcan midwife toad | *Alytes muletensis* | 6 | *-* |
| Lampila *et al.* 2009 | Siberian flying squirrel | *Pteromys volans* | 6 | 0.23 |
| Raeymaekers *et al.* 2009 | three-spined stickleback | *Gasterosteus aculeatus* | 6 | 0.069 |
| Keller *et al.* 2010 | dragonfly | *Leucorrhinia caudalis* | 6 | 0.13 |
| Beatty *et al.* 2011 | Red Squirrels | *Tamiasciurus hudsonicus* | 6 | *-* |
| Ruykys *et al.* 2015 | black-footed rock-wallaby | *Petrogale lateralis* | 6 | *-* |
| Montoya-Maya *et al.* 2016 | Coral | *Acropora austera* | 6 | 0.097 |
| Rivers *et al.* 2005 | Netterers bats | *Myotis -ttereri* | 7 | 0.017 |
| Crispo *et al.* 2006 | trinnidanian guppies | *Poecilia reticulata* | 7 | 0.302 |
| Piggott Banks & Taylor 2006 | brush-tailed rock-wallaby | *Petrogale penicillata* | 7 | 0.115 |
| Taylor and Costello 2006 | bull trout | *Salvelinus confluentus* | 7 | 0.46 |
| Maher 2009 | woodchuck | *Marmota mo-x* | 7 | *-* |
| Dubey & Shine 2010 | montane lizard | *Eulamprus leuraensis* | 7 | 0.128 |
| Cain Livieri & Swanson 2011 | footed ferrets | *Mustela nigripes* | 7 | 0.084 |
| Cardoso *et al.* 2014 | Eastern quoll | *Dasyurus viverrinus* | 7 | 0.131 |
| Dutta *et al.* 2015 | Sloth bear | *Melursus ursinus* | 7 | 0.042 |
| Oyler-McCance *et al.* 2005 | Gunnison sage-grouse | *Centrocercus minimus* | 8 | 0.2639 |
| Dupont Bourret & Ber*-*tchez 2007 | walleye | *Sander vitreus* | 8 | *-* |
| Underwood *et al.* 2007 | brooding coral | *-* | 8 | 0.095 |
| Vignieri 2007 | Pacific jumping mouse | *Zapus trinotatus* | 8 | 0.043 |
| Ciosi *et al.* 2008 | western corn rootworm | *Diabrotica virgifera virgifera* | 8 | 0.16 |
| Abdelkrim Pascal & Samadi 2009 | ship rat | *Rattus norvegicus* | 8 | *-* |
| Anderson & Meikle 2010 | mice | *Peromyscus leucopus* | 8 | 0.041 |
| Banks *et al.* 2010 | Sea Urchin | *Centrostephanus rodgersii* | 8 | 0.022 |
| Bronnenhuber *et al.* 2011 | round goby | *Neogobius melanostomus* | 8 | *-* |
| Hilmo *et al.* 2012 | epiphytic lichen | *Lobaria pulmo-ria* | 8 | 0.07 |
| Miralles *et al.* 2013 | Pilot whale | *Globicephala melas and Globicephala macrorhynchus* | 8 | 0.2957 |
| Wall *et al.* 2014 | Plant | *Astragalus michauxii* | 8 | 0.08 |
| Ceresa *et al.* 2015 | Moustached warbler | *Acrocephalis melanopogon* | 8 | *-* |
| Miller *et al.* 2015 | Maria*-* Common Moorhen | *Gallinula chloropus guami* | 8 | 0.152 |
| Mäder *et al.* 2016 | Fireweed | *Senecio madagascariensis* | 8 | 0.312 |
| Beneteau *et al.* 2008 | Greenside Darter | *Etheostoma Blennioides* | 9 | 0.076 |
| Bradbury, Campa*-* & Bentzen 2008 | rainbow smelt | *Osmerys mordax* | 9 | 0.053 |
| Cully & Hardiman. 2008 | or*-*mental pear tree | *Pyrus callerya-* | 9 | *-* |
| Janssens *et al.* 2008 | european otters | *Lutra lutra* | 9 | 0.102 |
| Cibrišn-Jaramillo *et al.* 2009 | fishtail palm | *Chamaedorea ernesti-august* | 9 | *-* |
| Sloop *et al.* 2010 | *-* | *Lim-nthes floccosa ssp. Califonica* | 9 | 0.65 |
| Kitanishi *et al.* 2012 | masu salmon | *Oncorhynchus masou* | 9 | 0.011 |
| Qi *et al.* 2013 | Qinghai toad-headed agamid | *Phrynocephalus vlangalii* | 9 | *-* |
| Sackett *et al.* 2013 | black-tailed prairie dog | *Cynomys ludovicianus* | 9 | 0.1136 |
| Sackett *et al.* 2013 | prarie dog | *Cynomys spp* | 9 | *-* |
| Dalen *et al.* 2006 | arctic fox | *Alopex lagopus* | 10 | *-* |
| Plath *et al.* 2007 | livebearing fish | *Poecilia mexica-* | 10 | 0.24 |
| Schweizer *et al.* 2007 | common vole | *Microtus arvalis* | 10 | *-* |
| Aspi *et al.* 2008 | Grey Wolf | *Canis lupus* | 10 | 0.152 |
| Marcet *et al.* 2008 | *-* | *Triatoma infestans* | 10 | 0.027 |
| Perez de Rosas *et al.* 2008 | Triatoma infestans | *Hemiptera: Reduviidae* | 10 | *-* |
| van Oppen *et al.* 2008 | coral | *Seriatopora hystrix* | 10 | 0.201 |
| Gauffre *et al.* 2009 | common vole | *Microtus arvalis* | 10 | *-* |
| Liu *et al.* 2009 | Yun*-*n snub-nosed monkeys | *Rhinopithecus bieti* | 10 | *-* |
| Noreen Harrison & Van Oppen 2009 | brooding reef coral | *Seriatopora hystrix* | 10 | 0.224 |
| Thibault *et al.* 2009 | rainbow trout | *Oncorhynchus mykiss* | 10 | 0.14 |
| Andrews *et al.* 2010 | spinner dolphins | *Stenella longirostris* | 10 | *-* |
| Plath *et al.* 2010 | Atlantic molly | *Poecilia mexica- Poeciliidae* | 10 | 0.215 |
| Wellband *et al.* 2012 | juvenile steelhead | *Oncorhynchus mykiss* | 10 | 0.008 |
| Garner *et al.* 2013 | Walleye | *Sander vitreus* | 10 | *-* |
| Kangas *et al.* 2013 | Finnish moose | *Alces alces* | 10 | 0.041 |
| Awad *et al.* 2014 | Fir tree | *Abies cilicica Carr.* | 10 | 0.0536 |
| Frankham *et al.* 2014 | Potoroid | *Potorous tridactylus* | 10 | *-* |
| Tsuchida *et al.* 2014 | Paper wasp | *Polistes chinensis anten-lis* | 10 | 0.145 |
| Tsuchida *et al.* 2014 | Paper wasp | *Polistes chinensis anten-lis* | 10 | 0.057 |
| Ginson *et al.* 2015 | Easter sand darter | *Ammocrypta pellucidda* | 10 | *-* |
| van Oppen *et al.* 2015 | Coral | *Acropora millepora* | 10 | 0.055 |
| Richards *et al.* 2016 | Giant barrel sponge | *Xestospongia muta* | 10 | 0.119 |
| Peakall & Lindenmayer 2006 | bush rats | *Rattus fuscipes* | 11 | 0.056 |
| Sonstebo *et al.* 2006 | brown trout | *Salmo trutta* | 11 | 0.095 |
| Bergl & Vigilant 2007 | Cross River gorilla | *Gorilla gorilla diehli* | 11 | *-* |
| He *et al.* 2009 | arillate pea | *Daviesia triflora* | 11 | 0.03 |
| Lopes *et al.* 2009 | Portuguese grapevine | *Vitis vinifera subsp. sylvestris* | 11 | *-* |
| *-* | common starling | *Sturnus vulgaris* | 11 | *-* |
| Lin *et al.* 2011 | Chinook salmon | *Oncorhynchus tshawytscha* | 11 | 0.009 |
| Harrisson *et al.* 2012 | superb fairy-wren | *Malurus cyaneus* | 11 | *-* |
| Leigh *et al.* 2012 | African wild dog | *Lycaon pictus* | 11 | *-* |
| Harris *et al.* 2013 | Lake trout | *Salvelinus -maycush* | 11 | 0.025 |
| Peterson *et al.* 2014 | Sockeye salmon | *Oncorhynchus nerka* | 11 | *-* |
| NemeshÕzi *et al.* 2016 | White-tailed eagles | *Haliaeetus albicilla* | 11 | *-* |
| Walsh *et al.* 2016 | Woodland Deer Mouse | *Peromyscus maniculatus gracilis* | 11 | *-* |
| Hazlitt *et al.* 2006 | brush-tailed rocked wallaby | *Petrogale penicillata* | 12 | *-* |
| Fabbri *et al.* 2007 | Italian wolf | *Canis lupus* | 12 | 0.09 |
| Lin *et al.* 2008 | sockeye salmon | *Oncorhynchus nerka* | 12 | *-* |
| Potter *et al.* 2012 | short-eared rock-wallaby | *Petrogale brachyotis* | 12 | *-* |
| Kopatz *et al.* 2014 | Northern Quropean brown bear | *Ursus arctos* | 12 | *-* |
| Choi *et al.* 2015 | Long-tailed goral | *-emorhedus caudatus* | 12 | *-* |
| Kelly *et al.* 2016 | Sonog sparrow | *Melospiza melodia* | 12 | *-* |
| Unger *et al.* 2016 | Easter hellbenders | *Cryptobranchus alleganiensis alleganiensis* | 12 | *-* |
| Dionne *et al.* 2008 | Atlantic salmon | *Salmo salar* | 13 | 0.051 |
| Hall *et al.* 2009 | marbled murrelet | *Brachyramphus marmoratus* | 13 | *-* |
| Steinfartz *et al.* 2009 | gallapagos igua*-* | *Amblyrhynchus cristatus* | 13 | 0.12 |
| Haag *et al.* 2010 | Atlantic Forest jaguars | *Panthera onca* | 13 | 0.089 |
| Bock *et al.* 2011 | Violet tunicate | *Botrylloides violaceus* | 13 | 0.173 |
| Lin *et al.* 2011 | Chum salmon | *Oncorhynchus keta* | 13 | 0.005 |
| Clarke *et al.* 2013 | Cutting Grass | *Gahnia trifida* | 13 | 0.476 |
| Monceau *et al.* 2013 | Ze*-*ida Dove | *Ze-ida aurita* | 13 | *-* |
| Sorenson *et al.* 2013 | Marlin | *Makaira nigricans* | 13 | 0.0099 |
| Green *et al.* 2014 | white-tailed deer | *Odocoileus virginianus* | 13 | *-* |
| Nowak *et al.* 2014 | Brown bears | *Ursus arctos* | 13 | *-* |
| Munguia-Vega *et al.* 2015 | Cortes Geoduck | *Panopea globosa* | 13 | 0.022 |
| Palstra OConnell & Russante 2007 | Atlantic salmon | *Salmo salar* | 14 | *-* |
| Gauthier *et al.* 2013 | striped bass | *Morone saxatilis* | 14 | 0.28 |
| NiedziaŠkowska *et al.* 2016 | Moose | *Alces alces* | 14 | *-* |
| Proctor *et al.* 2005 | grizzly bear | *Ursus arctos* | 15 | *-* |
| Pope *et al.* 2007 | Eurasian badger | *Meles meles* | 15 | *-* |
| Lancaster *et al.* 2011 | common ringtail possum | *Pseudocheirus peregrinus* | 15 | *-* |
| Proctor *et al.* 2012 | grizzly bear | *Ursus arctos* | 15 | *-* |
| Olsen *et al.* 2014 | Harbour seal | *Phoca vituli-* | 15 | *-* |
| Zgurski *et al.* 2014 | Collared pika | *Ochoto- collaris* | 15 | *-* |
| Chau *et al.* 2015 | Wasp | *Vespula pensylvanica* | 15 | *-* |
| Kitanishi *et al.* 2016 | masu salmon | *Oncorhynchus masou masou* | 15 | 0.0206 |
| Peery *et al.* 2010 | marbled murrelets | *Brachyramphus marmoratus* | 16 | *-* |
| Ceresa *et al.* 2015 | Eurasion reed warbler | *Acrocephalis scirpaceus* | 16 | *-* |
| Bonin *et al.* 2013 | Seals | *Arctocephalus (Arctophoca) gazel-la* | 17 | 0.014 |
| Stewart *et al.* 2013 | Leatherback turtles | *Dermochelys coriacea* | 17 | *-* |
| de Camargo *et al.* 2015 | Sao Paulo Marsh Antwren | *Formicivora paludicola* | 17 | 0.103 |
| Harris *et al.* 2015 | Northern Dolly Varden | *Salvelinus malma malma* | 17 | 0.099 |
| Hall *et al.* 2016 | Californian black rail | *Laterallus jamaicensis* | 17 | 0.018 |
| Wood *et al.* 2016 | Nile monitor | *Varanus niloticus* | 17 | *-* |
| Munshi-South *et al.* 2010 | white-footed mouse | *Peromyscus leucopus* | 18 | 0.071 |
| Savage *et al.* 2010 | southern long-toes salamander | *macrodactylum sigillatum* | 18 | 0.27 |
| Moore *et al.* 2013 | Arctic char | *Salvelinus alpinus* | 18 | 0.038 |
| Meerow *et al.* 2007 | Iris hexago*-* | *Iridaceae* | 19 | *-* |
| Jedrzejewski *et al.* 2005 | grey wolves | *Canis lupus* | 20 | 0.004 |
| Juarez *et al.* 2016 | Cougar | *Puma concolor* | 20 | *-* |
| Hamner *et al.* 2014 | Hectors dolphins | *Cephalorhynchus hectori* | 21 | *-* |
| Zalewski *et al.* 2016 | American mink | *Neovison vison* | 21 | *-* |
| Yang *et al.* 2015 | Thrips | *Frankliniella occidentalis* | 24 | *-* |
| Buchalski *et al.* 2015 | Peninsular bighorn sheep | *Ovis ca-densis nelsoni* | 25 | *-* |
| Ernest *et al.* 2014 | California puma | *Puma concolor* | 46 | *-* |
| Coleman *et al.* 2013 | Kelp | *Ekonia radiata* | *-* | *-* |
| Jensen *et al.* 2014 | Atlantic salmon | *salmon salar* | *-* | *-* |

References

Abdelkrim, J., Pascal, M., & Samadi, S. (2008). Genetic structure and functioning of alien ship rat populations from a Corsican micro-insular complex. *Biological Invasions*, *11*(3), 473–482. doi:10.1007/s10530-008-9263-9

Anderson, C. S., & Meikle, D. B. (2010). Genetic estimates of immigration and emigration rates in relation to population density and forest patch area in Peromyscus leucopus. *Conservation Genetics*, *11*(5), 1593–1605. doi:10.1007/s10592-009-0033-8

Andrews, K. R., Karczmarski, L., Au, W. W. L., Rickards, S. H., Vanderlip, C. A., Bowen, B. W., Gordon Grau, E., *et al.* (2010). Rolling stones and stable homes: social structure, habitat diversity and population genetics of the Hawaiian spinner dolphin (Stenella longirostris). *Molecular ecology*, *19*(4), 732–48. doi:10.1111/j.1365-294X.2010.04521.x

Aspi, J., Roininen, E., Kiiskilä, J., Ruokonen, M., Kojola, I., Bljudnik, L., Danilov, P., *et al.* (2008). Genetic structure of the northwestern Russian wolf populations and gene flow between Russia and Finland. *Conservation Genetics*, *10*(4), 815–826. doi:10.1007/s10592-008-9642-x

Awad, L., Fady, B., Khater, C., Roig, A., & Cheddadi, R. (2014) Genetic Structure and Diversity of the Endangered Fir Tree of Lebanon (*Abies cilicica* Carr.): Implications for Conservation. *PLoS ONE* 9(2): e90086. doi:10.1371/journal.pone.0090086

Banks, S. C., Ling, S. D., Johnson, C. R., Piggott, M. P., Williamson, J. E., & Beheregaray, L. B. (2010). Genetic structure of a recent climate change-driven range extension. *Molecular ecology*, *19*(10), 2011–24. doi:10.1111/j.1365-294X.2010.04627.x

Beatty, W. S., Liu, Z., Latch, E. K., & Nelson, T. A. (2011). Genetic assessment of the red squirrel in Illinois: Immigrants or Exotics? *The Jour-l of Wildlife Ma-gement*, *75*(5), 1236–1242. doi:10.1002/jwmg.156

Beneteau, C. L., Mandrak, N. E., & Heath, D. D. (2008). The effects of river barriers and range expansion of the population genetic structure and stability in Greenside Darter (Etheostoma blennioides) populations. *Conservation Genetics*, *10*(2), 477–487. doi:10.1007/s10592-008-9627-9

Bergl, R. A., & Vigilant, L. (2007). Genetic a*-*lysis reveals population structure and recent migration within the highly fragmented range of the Cross River gorilla (Gorilla gorilla diehli). *Molecular ecology*, *16*(3), 501–16. doi:10.1111/j.1365-294X.2006.03159.x

Bock, D. G., Zhan, A., Lejeusne, C., MacIsaac, H. J., & Cristescu, M. E. (2011). Looking at both sides of the invasion: patterns of colonization in the violet tunicate Botrylloides violaceus. *Molecular ecology*, *20*(3), 503–16. doi:10.1111/j.1365-294X.2010.04971.x

Bonanomi, S., Overgaard Therkildsen, N., Retzel, A., Berg Hedeholm, R., Pedersen, M. W., Meldrup, D., Pampoulie, C., Hemmer-Hansen, J., Grønkjær, P. and Nielsen, E. E. (2016), Historical DNA documents long-distance natal homing in marine fish. *Molecular Ecology*, 25: 2727–2734. doi: 10.1111/mec.13580

Bonin, C. A., Goebel, M. E., Forcada, J., Burton, R. S., & Hoffman, J. I. (2013). Unexpected genetic differentiation between recently recolonized populations of a long-lived and highly vagile marine mammal. *Ecology and Evolution*, 3(11), 3701–3712. http://doi.org/10.1002/ece3.732

Bradbury, I. R., Campa*-*, S. E., & Bentzen, P. (2008). Estimating contemporary early life-history dispersal in an estuarine fish: integrating molecular and otolith elemental approaches. *Molecular ecology*, *17*(6), 1438–50. doi:10.1111/j.1365-294X.2008.03694.x

Bronnenhuber, J., Dufour, B., Higgs, D., & Heath, D. (2011). Dispersal strategies, secondary range expansion and invasion genetics of the nonindigenous round goby, Neogobius melanostomus, in Great Lakes tributaries. *Molecular Ecology*, no–no. doi:citeulike-article-id:8962598

Buchalski, M. R., Navarro, A. Y., Boyce, W. M., Winston Vickers, T., Tobler, M. W., Nordstrom, L. A., ... Ernest, H. B. (2015). Genetic population structure of Peninsular bighorn sheep (*Ovis canadensis nelsoni*) indicates substantial gene flow across US-Mexico border. *Biological Conservation*, 184, 218-228. DOI: 10.1016/j.biocon.2015.01.006

Cain, C. M., Livieri, T. M., & Swanson, B. J. (2011). Genetic evaluation of a reintroduced population of black-footed ferrets (Mustela nigripes). *Jour-l of Mammalogy*, *92*(4), 751–759. doi:10.1644/10-MAMM-S-104.1

Cardoso, M. J., Mooney, N., Eldridge, M. D. B., Firestone, K. B., & Sherwin, W. B. (2014). Genetic monitoring reveals significant population structure in eastern quolls: implications for the conservation of a threatened carnivorous marsupials. *Australian Mammology*, 36(2), 169-177. doi:10.1071/AM13035

Ceresa, F., Belda, E. J., Kvist, L., Rguibi-Idrissi, H. and Monrós, J. S. (2015), Does fragmentation of wetlands affect gene flow in sympatric Acrocephalus warblers with different migration strategies?. *Journal of Avian Biol,* 46: 577–588. doi:10.1111/jav.00589

Chau, L. M., Hanna, C., Jenkins, L. T., Kutner, R. E., Burns, E. A., Kremen, C. & Goodisman, M. A. D. (2015), Population genetic structure of the predatory, social wasp *Vespula pensylvanica* in its native and invasive range. Ecology and Evolution, 5: 5573–5587. doi:10.1002/ece3.1757

Chevolot, M., Ellis, J. R., Hoarau, G., Rijnsdorp, A. D., Stam, W. T., & Olsen, J. L. (2006). Population structure of the thornback ray (Raja clavata L.) in British waters. *Jour-l of Sea Research*, *56*(4), 305–316. doi:10.1016/j.seares.2006.05.005

Choi, S. K., Chun, S. An, J., Lee, M-Y., Kim, H. J., Min, M-S. *et al.* (2015). Genetic diversity and population structure of the long-tailed goral, *Naimorhedus caudatus*, in South Korea. *Genes & Genetic Systems*, 90(1), 31-41. doi:10.1266/ggs.90.31

Cibrián-Jaramillo, A., Bacon, C. D., Garwood, N. C., Bateman, R. M., Thomas, M. M., Russell, S., Bailey, C. D., *et al.* (2009). Population genetics of the understory fishtail palm Chamaedorea ernesti-augusti in Belize: high genetic connectivity with local differentiation. *BMC genetics*, *10*(1), 65. doi:10.1186/1471-2156-10-65

Ciosi, M., Miller, N. J., Kim, K. S., Giordano, R., Estoup, A., & Guillemaud, T. (2008). Invasion of Europe by the western corn rootworm, Diabrotica virgifera virgifera: multiple transatlantic introductions with various reductions of genetic diversity. *Molecular ecology*, *17*(16), 3614–27. doi:10.1111/j.1365-294X.2008.03866.x

Clarke, L. J., Whalen, M. A. and Mackay, D. A. (2013), Cutting grass on desert islands: genetic structure of disjunct coastal and central Australian populations of *Gahnia trifida* (Cyperaceae). Journal Biogeography, 40: 1071–1081. doi:10.1111/jbi.12066

Coleman, M. A. (2013) Connectivity of the Habitat-Forming Kelp, *Ecklonia radiata* within and among Estuaries and Open Coast. *PLoS ONE* 8(5): e64667. doi:10.1371/journal.pone.0064667

Crispo, E., Bentzen, P., Reznick, D. N., Kinnison, M. T., & Hendry, A. P. (2006). The relative influence of *-*tural selection and geography on gene flow in guppies. *Molecular ecology*, *15*(1), 49–62. doi:10.1111/j.1365-294X.2005.02764.x

Culley, T. M., & Hardiman, N. A. (2008). The role of intraspecific hybridization in the evolution of invasiveness: a case study of the or*-*mental pear tree Pyrus callerya*-*. *Biological Invasions*, *11*(5), 1107–1119. doi:10.1007/s10530-008-9386-z

Dalén, L., Kvaløy, K., Linnell, J. D. C., Elmhagen, B., Strand, O., Tannerfeldt, M., Henttonen, H., *et al.* (2006). Population structure in a critically endangered arctic fox population: does genetics matter? *Molecular ecology*, *15*(10), 2809–19. doi:10.1111/j.1365-294X.2006.02983.x

de Camargo C, Gibbs HL, Costa MC, Del-Rio G, Silveira LF, Wasko AP, et al. (2015) Marshes as “Mountain Tops”: Genetic Analyses of the Critically Endangered São Paulo Marsh Antwren (Aves: Thamnophilidae). *PLoS ONE*, 10(10): e0140145. doi:10.1371/journal.pone.0140145

Dionne, M., Caron, F., Dodson, J. J., & Ber*-*tchez, L. (2008). Landscape genetics and hierarchical genetic structure in Atlantic salmon: the interaction of gene flow and local adaptation. *Molecular ecology*, *17*(10), 2382–96. doi:10.1111/j.1365-294X.2008.03771.x

Dubey, S., & Shine, R. (2010). Restricted dispersal and genetic diversity in populations of an endangered montane lizard (Eulamprus leuraensis, Scincidae). *Molecular ecology*, *19*(5), 886–97. doi:10.1111/j.1365-294X.2010.04539.x

Dupont, P.-P., Bourret, V., & Ber*-*tchez, L. (2007). Interplay between ecological, behavioural and historical factors in shaping the genetic structure of sympatric walleye populations (Sander vitreus). *Molecular ecology*, *16*(5), 937–51. doi:10.1111/j.1365-294X.2006.03205.x

Dutta T, Sharma S, Maldonado JE, Panwar HS, Seidensticker J (2015) Genetic Variation, Structure, and Gene Flow in a Sloth Bear (Melursus ursinus) Meta-Population in the Satpura-Maikal Landscape of Central India. *PLoS ONE* 10(5): e0123384. doi:10.1371/journal.pone.0123384

Ernest, H. B., Vickers, T. W., Morrison, S. A., Buchalski, M. R., & Boyce, W. M. (2014) Fractured Genetic Connectivity Threatens a Southern California Puma (*Puma concolor*) Population. *PLoS ONE* 9(10): e107985. doi:10.1371/journal.pone.0107985

Fabbri, E., Miquel, C., Lucchini, V., Santini, A., Caniglia, R., Duchamp, C., Weber, J.-M., *et al.* (2007). From the Apennines to the Alps: colonization genetics of the *-*turally expanding Italian wolf (Canis lupus) population. *Molecular ecology*, *16*(8), 1661–71. doi:10.1111/j.1365-294X.2007.03262.x

Frankham, G.J., Handasyde, K.A., Norton, M., Murray, A., & Eldridge, M. D. B. (2014). Molecular detection of intra-population structure in a threatened potoroid, *Potorous tridactylus*: conservation management and sampling implications. *Conservation Genetics*, 15. doi:10.1007/s10592-013-0560-1

Garner, S. R., Bobrowicz, S. M. and Wilson, C. C. (2013), Genetic and ecological assessment of population rehabilitation: walleye in Lake Superior. *Ecological Applications*, 23: 594–605. doi:10.1890/12-1099.1

Gauffre, B., Petit, E., Brodier, S., Bretagnolle, V., & Cosson, J. F. (2009). Sex-biased dispersal patterns depend on the spatial scale in a social rodent. *Proceedings. Biological sciences / The Royal Society*, *276*(1672), 3487–94. doi:10.1098/rspb.2009.0881

Gauthier, D. T., Audemard, C. A., Carlsson, J. E. L., Darden, T. L., Denson, M. R., Reece, K. S., & Carlsson, J. (2013). Genetic Population Structure of US Atlantic Coastal Striped Bass (Morone saxatilis). *Journal of Heredity*, (2013) 104(4): 510-520 doi:10.1093/jhered/est031

Ginson, R., Walter, R. P., Mandrak, N. E., Beneteau, C. L., & Heath, D. D. (2015). Hierarchical analysis of genetic structure in the habitat-specialist Eastern Sand Darter (*Ammocrypta pellucida*). *Ecology and Evolution*, 5(3), 695–708. http://doi.org/10.1002/ece3.1392

Graciá, E., Giménez, A., A*-*dón, J. D., Harris, D. J., Fritz, U., & Botella, F. (2012). The uncertainty of Late Pleistocene range expansions in the western Mediterranean: a case study of the colonization of south-eastern Spain by the spur-thighed tortoise, Testudo graeca. (B. Riddle, Ed.)*Jour-l of Biogeography*, n/a–n/a. doi:10.1111/jbi.12012

Green, M. L., Manjerovic, M. B., Pinilla, N. M., & Novakofski, J. (2014). Genetic assignment tests reveal dispersal of white-tailed deer: implications for chronic wasting disease. *Journal of Mammalogy,* 2014, 95 (3), 646-654

Haag, T., Santos, A. S., Sa*-*, D. A., Morato, R. G., Cullen, L., Crawshaw, P. G., De Angelo, C., *et al.* (2010). The effect of habitat fragmentation on the genetic structure of a top predator: loss of diversity and high differentiation among rem*-*nt populations of Atlantic Forest jaguars (Panthera onca). *Molecular ecology*, *19*(22), 4906–21. doi:10.1111/j.1365-294X.2010.04856.x

Hall, L. A., Palsbøll, P. J., Beissinger, S. R., Harvey, J. T., Bérubé, M., Raphael, M. G., Nelson, S. K., *et al.* (2009). Characterizing dispersal patterns in a threatened seabird with limited genetic structure. *Molecular ecology*, *18*(24), 5074–85. doi:10.1111/j.1365-294X.2009.04416.x

Hall, L. A. and Beissinger, S. R. (2016), Inferring the timing of long-distance dispersal between rail metapopulations using genetic and isotopic assignments. Ecological Applications. Accepted Author Manuscript. doi:10.1002/eap.1432

Hamner, R. M., Constantine, R., Oremus, M., Stanley, M., Brown, P. and Scott Baker, C. (2014), Long-range movement by Hector's dolphins provides potential genetic enhancement for critically endangered Maui's dolphin. Marine Mammal Science, 30: 139–153. doi:10.1111/mms.12026

Harris, L. N., Howland, K. L., Kowalchuk, M. W., Bajno, R., Lindsay, M. M., & Taylor, E. B. (2013). Microsatellite and mtDNA analysis of lake trout, *Salvelinus namaycush*, from Great Bear Lake, Northwest Territories: impacts of historical and contemporary evolutionary forces on Arctic ecosystems. *Ecology and Evolution*, 3(1), 145–161. http://doi.org/10.1002/ece3.439

Harris L. N., Bajno R., Gallagher, C. P., Koizumi, I., Johnson, L. K., Howland, K. L., Taylor, E. B., Reist, J. D. (2015). Life-history characteristics and landscape attributes as drivers of genetic variation, gene flow, and fine-scale population structure in northern Dolly Varden (*Salvelinus malma malma*) in Canada. Canadian Journal of Fisheries and Aquatic Sciences, 2015, 72:1477-1493, 10.1139/cjfas-2015-0016

Harrisson, K. A., Pavlova, A., Amos, J. N., Takeuchi, N., Lill, A., Radford, J. Q., & Sunnucks, P. (2012). Disrupted fine-scale population processes in fragmented landscapes despite large-scale genetic connectivity for a widespread and common cooperative breeder: the superb fairy-wren (Malurus cyaneus). *The Jour-l of animal ecology*. doi:10.1111/1365-2656.12007

Hazlitt, S. L., Goldizen, A. W., & Eldridge, M. D. B. (2006). Significant patterns of population genetic structure and limited gene flow in a threatened macropodid marsupial despite continuous habitat in southeast Queensland, Australia. *Conservation Genetics*, *7*(5), 675–689. doi:10.1007/s10592-005-9101-x

He, T., Lamont, B. B., Krauss, S. L., Enright, N. J., Miller, B. P., & Gove, A. D. (2009). Ants cannot account for interpopulation dispersal of the arillate pea Daviesia triflora. *The New phytologist*, *181*(3), 725–33. doi:10.1111/j.1469-8137.2008.02686.x

Hilmo, O., Lundemo, S., Holien, H., Stengrundet, K., & Stenøien, H. K. (2012). Genetic structure in a fragmented Northern Hemisphere rainforest: large effective sizes and high connectivity among populations of the epiphytic lichen Lobaria pulmo*-*ria. *Molecular ecology*, *21*(13), 3250–65. doi:10.1111/j.1365-294X.2012.05605.x

Jensen, A. J., Karlsson, S., Fiske, P., Hansen, L. P., Østborg, G. M., & Hindar, K. (2014). Origin and life history of Atlantic salmon (*Salmo salar*) near their northernmost oceanic limit. *Canadian Journal of Fisheries and Aquatic Sciences*, 71:1740-1746, doi:10.1139/cjfas-2014-0169

Janssens, X., Fontaine, M. C., Michaux, J. R., Libois, R., De Kermabon, J., Defourny, P., & Baret, P. V. (2008). Genetic pattern of the recent recovery of European otters in southern France. *Ecography*, *31*(2), 176–186. doi:10.1111/j.0906-7590.2008.4936.x

Jędrzejewski, W., Branicki, W., Veit, C., MeĐugorac, I., Pilot, M., Bunevich, A. N., Jędrzejewska, B., *et al.* (2005). Genetic diversity and relatedness within packs in an intensely hunted population of wolvesCanis lupus. *Acta Theriologica*, *50*(1), 3–22. doi:10.1007/BF03192614

Juarez, R.L., Schwartz, M.K., Pilgrim, K.L. et al. (2016). Assessing temporal genetic variation in a cougar population: influence of harvest and neighboring populations. *Conservation Genetics*, 17(2), 379-388. doi:10.1007/s10592-015-0790-5

Kangas, V. M., Kvist, L., Laaksonen, S., Nygren, T., & Aspi, J. (2013). Present genetic structure revealed by microsatellites reflects recent history of the Finnish moose (*Alces alces*). *European Journal of Wildlife Research*, 59: 613. doi:10.1007/s10344-013-0712-0

Keller, D., Brodbeck, S., Flöss, I., Vonwil, G., & Holderegger, R. (2010). Ecological and genetic measurements of dispersal in a threatened dragonfly. *Biological Conservation*, *143*(11), 2658–2663. doi:10.1016/j.biocon.2010.07.008

Kelly, T.R., MacGillivray, H.L., Sarquis-Adamson, Y. *et al*. (2016). Seasonal migration distance varies with natal dispersal and predicts parasitic infection in song sparrows. *Behavioral Ecology and Sociobiology*. 70: 1857. doi:10.1007/s00265-016-2191-2

Kitanishi, S., Yamamoto, T., Ishii, H., Yamaguchi, Y., & Kobayashi, T. (2016). Dispersal patterns of anadromous and freshwater resident masu salmon at different spatial scales in mid-western Hokkaido, Japan. *Ichthyological Research*. doi:10.1007/s10228-016-0525-8

Kitanishi, S., Yamamoto, T., Koizumi, I., Dunham, J. B., & Higashi, S. (2012). Fine scale relationships between sex, life history, and dispersal of masu salmon. *Ecology and evolution*, *2*(5), 920–9. doi:10.1002/ece3.228

Kopatz, A., Eiken, H. G., Aspi, J., Kojola, I., Tobiassen, C., Tirronen, K. F., et al. (2014) Admixture and Gene Flow from Russia in the Recovering Northern European Brown Bear (*Ursus arctos*). *PLoS ONE* 9(5). doi:10.1371/journal.pone.0097558

Kraaijeveld-Smit, F. J. L., Beebee, T. J. C., Griffiths, R. A., Moore, R. D., & Schley, L. (2005). Low gene flow but high genetic diversity in the threatened Mallorcan midwife toad Alytes muletensis. *Molecular ecology*, *14*(11), 3307–15. doi:10.1111/j.1365-294X.2005.02614.x

Lampila, S., Kvist, L., Wistbacka, R., & Orell, M. (2009). Genetic diversity and population differentiation in the endangered Siberian flying squirrel (Pteromys volans) in a fragmented landscape. *European Jour-l of Wildlife Research*, *55*(4), 397–406. doi:10.1007/s10344-009-0259-2

Lancaster, M. L., Taylor, A. C., Cooper, S. J. B., & Carthew, S. M. (2011). Limited ecological connectivity of an arboreal marsupial across a forest/plantation landscape despite apparent resilience to fragmentation. *Molecular ecology*, *20*(11), 2258–71. doi:10.1111/j.1365-294X.2011.05072.x

Leigh, K. A., Zenger, K. R., Tammen, I., & Raadsma, H. W. (2012). Loss of genetic diversity in an outbreeding species: small population effects in the African wild dog (Lycaon pictus). *Conservation Genetics*, *13*(3), 767–777. doi:10.1007/s10592-012-0325-2

Lin, J. E., Hilborn, R., Quinn, T. P., & Hauser, L. (2011). Self-sustaining populations, population sinks or aggregates of strays: chum (Oncorhynchus keta) and Chinook salmon (Oncorhynchus tshawytscha) in the Wood River system, Alaska. *Molecular ecology*, *20*(23), 4925–37. doi:10.1111/j.1365-294X.2011.05334.x

Lin, J., Quinn, T. P., Hilborn, R., & Hauser, L. (2008). Fine-scale differentiation between sockeye salmon ecotypes and the effect of phenotype on straying. *Heredity*, *101*(4), 341–50. doi:10.1038/hdy.2008.59

Liu, Z., Ren, B., Wu, R., Zhao, L., Hao, Y., Wang, B., Wei, F., *et al.* (2009). The effect of landscape features on population genetic structure in Yun*-*n snub-nosed monkeys (Rhinopithecus bieti) implies an anthropogenic genetic discontinuity. *Molecular ecology*, *18*(18), 3831–46. doi:10.1111/j.1365-294X.2009.04330.x

Lopes, M. S., Mendonça, D., Rodrigues dos Santos, M., Eiras-Dias, J. E., & Da Câmara Machado, A. (2009). New insights on the genetic basis of Portuguese grapevine and on grapevine domestication. *Genome / -tio-l Research Council Ca-da = Génome / Conseil -tio-l de recherches Ca-da*, *52*(9), 790–800. doi:10.1139/g09-048

Mäder, G., Castro, L., Bonatto, S. L., & de Freitas, L. B. (2016). Multiple introductions and gene flow in subtropical South American populations of the fireweed, Senecio madagascariensis(Asteraceae). *Genetics and Molecular Biology*, 39(1), 135–144. http://doi.org/10.1590/1678-4685-GMB-2015-0167

Maher, C. R. (2009). Genetic relatedness and space use in a behaviorally flexible species of marmot, the woodchuck (Marmota mo*-*x). *Behavioral Ecology and Sociobiology*, *63*(6), 857–868. doi:10.1007/s00265-009-0726-5

Marcet, P. L., Mora, M. S., Cutrera, A. P., Jones, L., Gürtler, R. E., Kitron, U., & Dotson, E. M. (2008). Genetic structure of Triatoma infestans populations in rural communities of Santiago del Estero, northern Argenti*-*. *Infection, genetics and evolution : jour-l of molecular epidemiology and evolutio-ry genetics in infectious diseases*, *8*(6), 835–46. doi:10.1016/j.meegid.2008.08.002

Meerow, A. W., Gideon, M., Kuhn, D. N., Motamayor, J. C., & Kamura, K. (2007). Genetic Structure and Gene Flow among South Florida Populations of Iris hexago*-* Walt. (Iridaceae) Assessed with 19 Microsatellite D*-* Loci. *Inter-tio-l Jour-l of Plant Sciences*, *168*(9), 1291–1309. doi:10.1086/521692

Miller, M. P., Mullins, T. D., Haig , S. M., Takano, L., & Garcia, K. (2015). Genetic structure, diversity, and interisland dispersal in the endangered Mariana Common Moorhen (*Gallinula chloropus guami*). *The Condor*, 117(4), 660-669. doi:10.1650/CONDOR-15-42.1

Miralles L, Lens S, Rodríguez-Folgar A, Carrillo M, Martín V, Mikkelsen B, et al. (2013) Interspecific Introgression in Cetaceans: DNA Markers Reveal Post-F1 Status of a Pilot Whale. *PLoS ONE* 8(8): e69511. doi:10.1371/journal.pone.0069511

Monceau K, Cézilly F, Moreau J, Motreuil S, Wattier R (2013) Colonisation and Diversification of the Zenaida Dove (*Zenaida aurita*) in the Antilles: Phylogeography, Contemporary Gene Flow and Morphological Divergence. *PLoS ONE* 8(12), doi:10.1371/journal.pone.0082189

Montoya-Maya, P.H., Schleyer, M.H. & Macdonald, A.H.H. (2016). Limited ecologically relevant genetic connectivity in the south-east African coral populations calls for reef-level management. *Marine Biology*, 163. doi:10.1007/s00227-016-2939-2

Moore, J-S., Harris, L. N., Tallman, R. F., & Taylor, E. B. (2013). The interplay between dispersal and gene flow in anadromous Arctic char (*Salvelinus alpinus*): implications for potential for local adaptation. *Canadian Journal of Fisheries and Aquatic Sciences*, 70:1327-1338, 10.1139/cjfas-2013-0138

Munguia-Vega, A., Leyva-Valencia, I., Lluch-Cota, D. B., and Cruz-Hernandez, P. (2015). Genetic Structure of the Cortes Geoduck *Panopea globosa* Dall, 1898, from the Mexican Northwest. *Journal of Shellfish Research*, 34 (1), 153-161

Munshi-South, J., & Kharchenko, K. (2010). Rapid, pervasive genetic differentiation of urban white-footed mouse (Peromyscus leucopus) populations in New York City. *Molecular ecology*. doi:10.1111/j.1365-294X.2010.04816.x

Nemesházi, E., Kövér, S., Zachos, F. E., Horváth, Z., Tihanyi, G., Mórocz, A., Mikuska, T., Hám, I., Literák, I., Ponnikas, S., Mizera, T. and Szabó, K. (2016), Natural and anthropogenic influences on the population structure of white-tailed eagles in the Carpathian Basin and central Europe. Journal of Avian Biology, 47: 795–805. doi:10.1111/jav.00938

Niedziałkowska, M., Jędrzejewska, B., Danyłow, J. & Niedzialkowski, K. (2016). Diverse rates of gene flow and long-distance migration in two moose *Alces alces* subpopulations in Europe. *Mammal Research*, 61. doi:10.1007/s13364-016-0274-0

Noreen, A. M. E., Harrison, P. L., & Van Oppen, M. J. H. (2009). Genetic diversity and connectivity in a brooding reef coral at the limit of its distribution. *Proceedings. Biological sciences / The Royal Society*, *276*(1675), 3927–35. doi:10.1098/rspb.2009.1050

Nowak, C., Domokos, C., Dutsov, A., & Frosch, C. (2014). Molecular evidence for historic long-distance translocations of brown bears in the Balkan region. *Conservation Genetics*, 15: 743. doi:10.1007/s10592-014-0570-7

Olsen, M. T., Andersen, L. W., Dietz, R., Teilmann, J., Härkönen, T. and Siegismund, H. R. (2014), Integrating genetic data and population viability analyses for the identification of harbour seal (*Phoca vitulina*) populations and management units. *Molecular Ecology*, 23: 815–831. doi:10.1111/mec.12644

Oyler-McCance, S. J., St. John, J., Taylor, S. E., Apa, A. D., & Quinn, T. W. (2005). POPULATION GENETICS OF GUNNISON SAGE-GROUSE: IMPLICATIONS FOR MA*-*GEMENT. *Jour-l of Wildlife Ma-gement*, *69*(2), 630–637. doi:10.2193/0022-541X(2005)069[0630:PGOGSI]2.0.CO;2

Palstra, F. P., O’Connell, M. F., & Ruzzante, D. E. (2007). Population structure and gene flow reversals in Atlantic salmon (Salmo salar) over contemporary and long-term temporal scales: effects of population size and life history. *Molecular ecology*, *16*(21), 4504–22. doi:10.1111/j.1365-294X.2007.03541.x

Peakall, R., & Lindenmayer, D. (2006). Genetic insights into population recovery following experimental perturbation in a fragmented landscape. *Biological Conservation*, *132*(4), 520–532. doi:10.1016/j.biocon.2006.05.013

Peery, M. Z., Hall, L. A., Sellas, A., Beissinger, S. R., Moritz, C., Bérubé, M., Raphael, M. G., *et al.* (2010). Genetic a*-*lyses of historic and modern marbled murrelets suggest decoupling of migration and gene flow after habitat fragmentation. *Proceedings. Biological sciences / The Royal Society*, *277*(1682), 697–706. doi:10.1098/rspb.2009.1666

Pérez de Rosas, A. R., Segura, E. L., Fichera, L., & García, B. A. (2008). Macrogeographic and microgeographic genetic structure of the Chagas’ disease vector Triatoma infestans (Hemiptera: Reduviidae) from Catamarca, Argenti*-*. *Genetica*, *133*(3), 247–60. doi:10.1007/s10709-007-9208-8

Peterson, D. A., Hilborn, R., & Hauser, L. (2014). Local adaptation limits lifetime reproductive success of dispersers in a wild salmon metapopulation. Nature Communications, 3696, doi:10.1038/ncomms4696

Piggott, M. P., Banks, S. C., & Taylor, A. C. (2006). Population structure of brush-tailed rock-wallaby (Petrogale penicillata) colonies inferred from a*-*lysis of faecal D*-*. *Molecular ecology*, *15*(1), 93–105. doi:10.1111/j.1365-294X.2005.02784.x

Plath, M, Hauswaldt, J. S., Moll, K., Tobler, M., García De León, F. J., Schlupp, I., & Tiedemann, R. (2007). Local adaptation and pronounced genetic differentiation in an extremophile fish, Poecilia mexica*-*, inhabiting a Mexican cave with toxic hydrogen sulphide. *Molecular ecology*, *16*(5), 967–76. doi:10.1111/j.1365-294X.2006.03212.x

Plath, Martin, Hermann, B., Schröder, C., Riesch, R., Tobler, M., García de León, F. J., Schlupp, I., *et al.* (2010). Locally adapted fish populations maintain small-scale genetic differentiation despite perturbation by a catastrophic flood event. *BMC evolutio-ry biology*, *10*(1), 256. doi:10.1186/1471-2148-10-256

Pope, L. C., Butlin, R. K., Wilson, G. J., Woodroffe, R., Erven, K., Conyers, C. M., Franklin, T., *et al.* (2007). Genetic evidence that culling increases badger movement: implications for the spread of bovine tuberculosis. *Molecular ecology*, *16*(23), 4919–29. doi:10.1111/j.1365-294X.2007.03553.x

Potter, S., Eldridge, M. D. B., Cooper, S. J. B., Paplinska, J. Z., & Taggart, D. A. (2012). Habitat connectivity, more than species’ biology, influences genetic differentiation in a habitat specialist, the short-eared rock-wallaby (Petrogale brachyotis). *Conservation Genetics*, *13*(4), 937–952. doi:10.1007/s10592-012-0342-1

Proctor, M. F., McLellan, B. N., Strobeck, C., & Barclay, R. M. R. (2005). Genetic a*-*lysis reveals demographic fragmentation of grizzly bears yielding vulnerably small populations. *Proceedings. Biological sciences / The Royal Society*, *272*(1579), 2409–16. doi:10.1098/rspb.2005.3246

Proctor, M. F., Paetkau, D., Mclellan, B. N., Stenhouse, G. B., Kendall, K. C., Mace, R. D., Kasworm, W. F., *et al.* (2012). Population fragmentation and inter-ecosystem movements of grizzly bears in western Ca*-*da and the northern United States. *Wildlife Monographs*, *180*(1), 1–46. doi:10.1002/wmon.6

Qi, Y., Yang, W., Lu, B., & Fu, J. (2013). Genetic evidence for male-biased dispersal in the Qinghai toad-headed agamid Phrynocephalus vlangalii and its potential link to individual social interactions. *Ecology and Evolution*, 3(5), 1219–1230. http://doi.org/10.1002/ece3.532

Raeymaekers, J. A. M., Raeymaekers, D., Koizumi, I., Geldof, S., & Volckaert, F. A. M. (2009). Guidelines for restoring connectivity around water mills: a population genetic approach to the ma*-*gement of riverine fish. *Jour-l of Applied Ecology*, *46*(3), 562–571. doi:10.1111/j.1365-2664.2009.01652.x

Richards, V. P., Bernard, A. M., Feldheim, K. A., & Shivji, M. S.(2016). Patterns of population structure and dispersal in the long-lived ‘‘redwood’’ of the coral reef, the giant barrel sponge (*Xestospongia muta*). Coral Reefs, doi:10.1007/s00338-016-1435-y

Rivers, N. M., Butlin, R. K., & Altringham, J. D. (2005). Genetic population structure of *-*tterer’s bats explained by mating at swarming sites and philopatry. *Molecular ecology*, *14*(14), 4299–312. doi:10.1111/j.1365-294X.2005.02748.x

Robinet, C., Imbert, C.-E., Rousselet, J., Sauvard, D., Garcia, J., Goussard, F., & Roques, A. (2011). Human-mediated long-distance jumps of the pine processio*-*ry moth in Europe. *Biological Invasions*, *14*(8), 1557–1569. doi:10.1007/s10530-011-9979-9

Rollins, L. A., Woolnough, A. P., Wilton, A. N., Sinclair, R., & Sherwin, W. B. (2009). Invasive species can’t cover their tracks: using microsatellites to assist ma*-*gement of starling ( *Sturnus vulgaris* ) populations in Western Australia. *Molecular Ecology*, *18*(8), 1560–1573. Retrieved from http://library.deakin.edu.au/resserv?sid=google&auinit=LEEANN&aulast=ROLLINS&atitle=Invasive+species+can’t+cover+their+tracks:+using+microsatellites+to+assist+ma*-*gement+of+starling+(Sturnus+vulgaris)+populations+in+Western+Australia&id=doi:10.1111/j.1365-294X.2009.04132.x&title=molecular+ecology&volume=18&issue=8&date=2009&spage=1560&issn=0962-1083

Rugman-Jones, P. F., Hoddle, M. S., & Stouthamer, R. (2007). Population genetics of Scirtothrips perseae: tracing the origin of a recently introduced exotic pest of Californian avocado orchards, using mitochondrial and microsatellite D*-* markers. *Entomologia Experimentalis et Applicata*, *124*(1), 101–115. doi:10.1111/j.1570-7458.2007.00559.x

Ruykys, L. & Lancaster, M. L. (2015). Population structure and genetic diversity of the black-footed rock-wallaby (Petrogale lateralis MacDonnell Ranges race). *Australian Journal of Zoology*, 63(2), 91-100. doi:10.1071/ZO14009

Sackett, L. C., Collinge, S. K., & Martin, A. P. (2013). Do pathogens reduce genetic diversity of their hosts? Variable effects of sylvatic plague in black-tailed prairie dogs. *Molecular ecology*. doi:10.1111/mec.12270

Savage, W. K., Fremier, A. K., & Shaffer, H. B. (2010). Landscape genetics of alpine Sierra Nevada salamanders reveal extreme population subdivision in space and time. *Molecular ecology*, *19*(16), 3301–14. doi:10.1111/j.1365-294X.2010.04718.x

Schweizer, M., Excoffier, L., & Heckel, G. (2007). Fine-scale genetic structure and dispersal in the common vole (Microtus arvalis). *Molecular ecology*, *16*(12), 2463–73. doi:10.1111/j.1365-294X.2007.03284.x

Scott, K. D., Lawrence, N., Lange, C. L., Scott, L. J., Wilkinson, K. S., Merritt, M. A., Miles, M., *et al.* (2005). Assessing Moth Migration and Population Structuring in Helicoverpa armigera (Lepidoptera: Noctuidae) at the Regio*-*l Scale: Example from the Darling Downs, Australia. *Jour-l of Economic Entomology*, *98*(6), 2210–2219. doi:10.1603/0022-0493-98.6.2210

Sloop, C. M., Pickens, C., & Gordon, S. P. (2010). Conservation genetics of Butte County meadowfoam (Lim*-*nthes floccosa ssp. californica Arroyo), an endangered ver*-*l pool endemic. *Conservation Genetics*, *12*(1), 311–323. doi:10.1007/s10592-010-0142-4

Sønstebø, J. H., Borgstrøm, R., & Heun, M. (2006). Genetic structure of brown trout (Salmo trutta L.) from the Hardangervidda mountain plateau (Norway) a*-*lyzed by microsatellite D*-*: a basis for conservation guidelines. *Conservation Genetics*, *8*(1), 33–44. doi:10.1007/s10592-006-9145-6

Sorenson, L., McDowell, J.R., Knott, T. & Graves, T. K. (2013). Assignment test method using hypervariable markers for blue marlin (*Makaira nigricans*) stock identification. *Conservation Genetic Resources*, 5, doi:10.1007/s12686-012-9747-x

Steinfartz, S., Glaberman, S., Lanterbecq, D., Russello, M. A., Rosa, S., Hanley, T. C., Marquez, C., *et al.* (2009). Progressive colonization and restricted gene flow shape island-dependent population structure in Galápagos marine igua*-*s (Amblyrhynchus cristatus). *BMC evolutio-ry biology*, *9*(1), 297. doi:10.1186/1471-2148-9-297

Stewart, K. R., James, M. C., Roden, S. and Dutton, P. H. (2013), Assignment tests, telemetry and tag-recapture data converge to identify natal origins of leatherback turtles foraging in Atlantic Canadian waters. Journal of Animal Ecology, 82: 791–803. DOI:10.1111/1365-2656.12056doi:10.1111/1365-2656.12056

Taylor, E. B., & Costello, A. B. (2006). Microsatellite D*-* a*-*lysis of coastal populations of bull trout ( Salvelinus confluentus ) in British Columbia: zoogeographic implications and its application to recreatio*-*l fishery ma*-*gement. *Ca-dian Jour-l of Fisheries and Aquatic Sciences*, *63*(5), 1157–1171. doi:10.1139/f06-026

Thibault, I., Ber*-*tchez, L., & Dodson, J. J. (2009). The contribution of newly established populations to the dy*-*mics of range expansion in a one-dimensio*-*l fluvial-estuarine system: rainbow trout ( Oncorhynchus mykiss ) in Eastern Quebec. *Diversity and Distributions*, *15*(6), 1060–1072. doi:10.1111/j.1472-4642.2009.00606.x

Tsuchida, K., Kudô, K. and Ishiguro, N. (2014), Genetic structure of an introduced paper wasp, *Polistes chinensis antennalis* (Hymenoptera, Vespidae) in New Zealand. *Molecular Ecology*, 23: 4018–4034. doi:10.1111/mec.12852

Underwood, J. N., Smith, L. D., Van Oppen, M. J. H., & Gilmour, J. P. (2007). Multiple scales of genetic connectivity in a brooding coral on isolated reefs following catastrophic bleaching. *Molecular ecology*, *16*(4), 771–84. doi:10.1111/j.1365-294X.2006.03187.x

Unger, S. D., Chapman, E. J., Regester, K. J., & Williams, R. N. (2016). Genetic signatures follow dendritic patterns in the eastern hellbender (*Cryptobrancus alleganiensis alleganiensis*). *Herpetological Conservation and Biology*, 11(1), 40-51

van Oppen, M. J. H., Lutz, A., De’ath, G., Peplow, L., & Kininmonth, S. (2008). Genetic traces of recent long-distance dispersal in a predomi*-*ntly self-recruiting coral. (S. Rands, Ed.)*PloS one*, *3*(10), e3401. doi:10.1371/jour*-*l.pone.0003401

van Oppen, M. J. H., Lukoschek, V., Berkelmans, R., Peplow, L. M., & Jones, A. M. (2015) A population genetic assessment of coral recovery on highly disturbed reefs of the Keppel Island archipelago in the southern Great Barrier Reef. *PeerJ* 3:e1092 doi:10.7717/peerj.1092

Vignieri, S. N. (2007). Cryptic behaviours, inverse genetic landscapes, and spatial avoidance of inbreeding in the Pacific jumping mouse. *Molecular ecology*, *16*(4), 853–66. doi:10.1111/j.1365-294X.2006.03182.x

Wall, W.A., Douglas, N.A., Hoffmann, W.A. et al. (2014). Evidence of population bottleneck in *Astragalus michauxii* (Fabaceae), a narrow endemic of the southestern United States. *Conserv Genetics* 15. doi:10.1007/s10592-013-0527-2

Walsh, S. E., Woods, W. E., and Hoffman, S. M. G. (2016). Effects of Range Contraction and Habitat Fragmentation on Genetic Variation in the Woodland Deer Mouse (*Peromyscus maniculatus gracilis*). *The American Midland Naturalist*, 176(2), 272-281. doi: 10.1674/0003-0031-176.2.272

Wellband, K. W., Atagi, D. Y., Koehler, R. A., & Heath, D. D. (2012). Fine-Scale Population Genetic Structure and Dispersal of Juvenile Steelhead in the Bulkley-Morice River, British Columbia. *Transactions of the American Fisheries Society*, *141*(2), 392–401. doi:10.1080/00028487.2012.667040

Wheat, C. W., Fescemyer, H. W., Kvist, J., Tas, E., Vera, J. C., Frilander, M. J., Hanski, I., *et al.* (2011). Functio*-*l genomics of life history variation in a butterfly metapopulation. *Molecular ecology*, *20*(9), 1813–28. doi:10.1111/j.1365-294X.2011.05062.x

Wilson, C. C., Lavender, M., & Black, J. (2007). Genetic Assessment of Walleye (Sander vitreus) Restoration Efforts and Options in Nipigon Bay and Black Bay, Lake Superior. *Jour-l of Great Lakes Research*, *33*(sp1), 133–144. doi:10.3394/0380-1330(2007)33[133:GAOWSV]2.0.CO;2

Yang, X.-M., Lou, H., Sun, J.-T., Zhu, Y.-M., Xue, X.-F., & Hong, X.-Y. (2015). Temporal Genetic Dynamics of an Invasive Species, Frankliniella occidentalis (Pergande), in an Early Phase of Establishment. *Scientific Reports*, 5, 11877. http://doi.org/10.1038/srep11877

Zalewski A, Zalewska H, Lunneryd S-G, André C, Mikusiński G (2016) Reduced Genetic Diversity and Increased Structure in American Mink on the Swedish Coast following Invasive Species Control. *PLoS ONE* 11(6): e0157972. doi:10.1371/journal.pone.0157972

Zgurski, J.M. & Hik, D.S. (2014). Gene flow and the restoration of genetic diversity in a fluctuating collared pika (*Ochotona collaris*) population. *Conservation Genet*ics. 15. doi:10.1007/s10592-013-0519-2

# Simulated Disperser A*-*lysis: determining the number of loci required to genetically identify dispersers

Adam P.A. Cardilini^1^, Craig D.H. Sherman^2^, William B. Sherwin^3^, Lee A. Rollins^2^

^1^ Faculty of Science, Engineering and Built Environment, Deakin University, 221 Burwood Hwy, Burwood, Victoria 3125, Australia

^2^ Centre for Integrative Ecology, School of Life and Environmental Science, Deakin University, 75 Pigdons Rd, Waurn Ponds, Victoria 3216, Australia

^3^ School of Biological, Earth and Environmental Sciences, The University of New South Wales, High St, Kensington, NSW 2052, Australia

Keywords: Ecological Genetics, Population Genetics, Power A*-*lysis, Migrant, GeneClass2

Corresponding Author:

Adam P.A. Cardilini

Faculty of Science, Engineering and Built Environment, Deakin University, 221 Burwood Hwy, Burwood, Victoria 3125, Australia

Email: a.cardilini@gmail.com
