# Supplementary material for: Simulated Disperser Analysis: determining the number of loci required to genetically identify dispersers
Source: PeerJ. 2018 Mar 29;6:e4573. doi: 10.7717/peerj.4573 (PMC5878929; doi:10.7717/peerj.4573)
Supplement: Table S3 — Hardy-Weinberg Equilibrium testing for the 20 markers described in this study (Table S1). Loci in bold show significant departures from Hardy-Weinberg equilibrium in the given population. Loci Svu002 and Svu010 have diverged from Hardy-Weinberg equilibrium in two populations and were removed from further analysis. Included for each population are: the population name locus name, the number of individuals genotyped for that locus, the observed and expected heterozygosity with standard deviations. Significant departures from Hardy-Weinberg Equilibrium are highlighted in bold. [file peerj-06-4573-s003.docx]

Supplementary table 3: Hardy-Weinberg Equilibrium testing for the 20 markers described in this study (Supplementary Table 1). Loci in bold show significant departures from Hardy-Weinberg equilibrium in the given population. Loci Svu002 and Svu010 have diverged from Hardy-Weinberg equilibrium in two populations and were removed from further analysis. Included for each population are: the population name locus name, the number of individuals genotyped for that locus, the observed and expected heterozygosity with standard deviations. Significant departures from Hardy-Weinberg Equilibrium are highlighted in bold.

| Population | Locus | No. indiv. genotyped | Obs. Het. | Exp. Het. | *P*-value | s.d. |
| --- | --- | --- | --- | --- | --- | --- |
| Munglinup | *Svu002* | 30 | 0.5 | 0.69661 | 0.0144 | 0.00011 |
|  | *Svu038* | 30 | 0.63333 | 0.67401 | 0.75755 | 0.00043 |
|  | *Svu004* | 29 | 0.55172 | 0.55838 | 0.17236 | 0.00039 |
|  | *Svu006* | 30 | 0.36667 | 0.38192 | 0.29365 | 0.00034 |
|  | *Svu007* | 30 | 0.53333 | 0.66215 | 0.40732 | 0.00046 |
|  | *Svu039* | 30 | 0.36667 | 0.43785 | 0.21296 | 0.00045 |
|  | *Svu005* | 30 | 0.63333 | 0.58192 | 0.02438 | 0.00016 |
|  | ***Svu033*** | **30** | **0.26667** | **0.65085** | **0** | **0** |
|  | *Svu013* | 30 | 0.6 | 0.83277 | 0.00909 | 0.00011 |
|  | *Svu018* | 30 | 0.63333 | 0.51808 | 0.26638 | 0.00051 |
|  | *Svu010* | 27 | 0.44444 | 0.72397 | 0.00365 | 0.00005 |
|  | *Svu030* | 30 | 0.5 | 0.52486 | 0.21535 | 0.0004 |
|  | *Svu012* | 30 | 0.53333 | 0.48927 | 0.93666 | 0.00025 |
|  | *Svu015* | 30 | 0.53333 | 0.68418 | 0.00918 | 0.0001 |
|  | *Svu017* | 29 | 0.68966 | 0.75197 | 0.00145 | 0.00004 |
|  | *Svu021* | 30 | 0.63333 | 0.59548 | 0.50063 | 0.00043 |
|  | *Svu022* | 27 | 0.77778 | 0.80363 | 0.84619 | 0.00036 |
|  | *Svu034* | 30 | 0.33333 | 0.41695 | 0.12909 | 0.00033 |
|  | *Svu016* | 30 | 0.63333 | 0.77062 | 0.1262 | 0.00034 |
|  | *Svu025* | 30 | 0.7 | 0.73955 | 0.00061 | 0.00002 |
| Mallala | ***Svu002*** | **31** | **0.51613** | **0.8477** | **0.0001** | **0.00001** |
|  | *Svu038* | 32 | 0.71875 | 0.79663 | 0.6911 | 0.00049 |
|  | *Svu004* | 32 | 0.65625 | 0.78968 | 0.18813 | 0.00041 |
|  | *Svu006* | 32 | 0.90625 | 0.89236 | 0.62038 | 0.00046 |
|  | *Svu007* | 32 | 0.53125 | 0.82887 | 0.00434 | 0.00006 |
|  | *Svu039* | 32 | 0.5 | 0.54514 | 0.56379 | 0.00059 |
|  | *Svu005* | 32 | 0.71875 | 0.63938 | 0.14278 | 0.00034 |
|  | *Svu033* | 32 | 0.6875 | 0.79613 | 0.07548 | 0.00027 |
|  | *Svu013* | 30 | 0.6 | 0.80621 | 0.00369 | 0.00005 |
|  | *Svu018* | 32 | 0.9375 | 0.81845 | 0.0558 | 0.00018 |
|  | ***Svu010*** | **28** | **0.32143** | **0.81234** | **0.00001** | **0** |
|  | *Svu030* | 32 | 0.625 | 0.66617 | 0.07261 | 0.00024 |
|  | *Svu012* | 32 | 0.8125 | 0.77034 | 0.51278 | 0.0005 |
|  | *Svu015* | 32 | 0.59375 | 0.76339 | 0.07956 | 0.00033 |
|  | *Svu017* | 30 | 0.66667 | 0.8435 | 0.12016 | 0.00022 |
|  | *Svu021* | 32 | 0.6875 | 0.57937 | 0.48389 | 0.00048 |
|  | *Svu022* | 31 | 0.96774 | 0.84981 | 0.07002 | 0.00026 |
|  | *Svu034* | 32 | 0.78125 | 0.7996 | 0.10447 | 0.00031 |
|  | *Svu016* | 31 | 0.67742 | 0.74299 | 0.68768 | 0.00048 |
|  | *Svu025* | 32 | 0.71875 | 0.80109 | 0.03408 | 0.00019 |
| Orange | ***Svu002*** | **31** | **0.32258** | **0.87097** | **0** | **0** |
|  | *Svu038* | 32 | 0.625 | 0.80903 | 0.02388 | 0.00015 |
|  | *Svu004* | 32 | 0.6875 | 0.8254 | 0.46227 | 0.00045 |
|  | *Svu006* | 32 | 0.90625 | 0.85615 | 0.92005 | 0.00019 |
|  | *Svu007* | 32 | 0.6875 | 0.87401 | 0.19405 | 0.00038 |
|  | *Svu039* | 32 | 0.5625 | 0.60565 | 0.85983 | 0.00034 |
|  | *Svu005* | 32 | 0.78125 | 0.74851 | 0.45656 | 0.00034 |
|  | *Svu033* | 32 | 0.625 | 0.82788 | 0.00218 | 0.00004 |
|  | ***Svu013*** | **31** | **0.51613** | **0.80169** | **0** | **0** |
|  | *Svu018* | 32 | 0.8125 | 0.79018 | 0.64891 | 0.00036 |
|  | ***Svu010*** | **25** | **0.2** | **0.78694** | **0** | **0** |
|  | *Svu030* | 32 | 0.65625 | 0.65079 | 0.3913 | 0.00051 |
|  | *Svu012* | 32 | 0.78125 | 0.77976 | 0.29739 | 0.00035 |
|  | *Svu015* | 32 | 0.46875 | 0.69048 | 0.00318 | 0.00006 |
|  | *Svu017* | 29 | 0.68966 | 0.89716 | 0.01719 | 0.00009 |
|  | *Svu021* | 32 | 0.90625 | 0.75347 | 0.55757 | 0.0005 |
|  | *Svu022* | 31 | 0.80645 | 0.82813 | 0.91703 | 0.00024 |
|  | *Svu034* | 32 | 0.78125 | 0.86954 | 0.0988 | 0.0002 |
|  | *Svu016* | 32 | 0.65625 | 0.85069 | 0.1157 | 0.00028 |
|  | *Svu025* | 32 | 0.6875 | 0.81845 | 0.44426 | 0.00047 |

# Simulated Disperser Analysis: determining the number of loci required to genetically identify dispersers

Adam P.A. Cardilini^1^, Craig D.H. Sherman^2^, William B. Sherwin^3^, Lee A. Rollins^2^

^1^ Faculty of Science, Engineering and Built Environment, Deakin University, 221 Burwood Hwy, Burwood, Victoria 3125, Australia

^2^ Centre for Integrative Ecology, School of Life and Environmental Science, Deakin University, 75 Pigdons Rd, Waurn Ponds, Victoria 3216, Australia

^3^ School of Biological, Earth and Environmental Sciences, The University of New South Wales, High St, Kensington, NSW 2052, Australia

Keywords: Ecological Genetics, Population Genetics, Power Analysis, Migrant, GeneClass2

Corresponding Author:

Adam P.A. Cardilini

Faculty of Science, Engineering and Built Environment, Deakin University, 221 Burwood Hwy, Burwood, Victoria 3125, Australia

Email: a.cardilini@gmail.com
