# Supplementary material for: Simulated Disperser Analysis: determining the number of loci required to genetically identify dispersers
Source: PeerJ. 2018 Mar 29;6:e4573. doi: 10.7717/peerj.4573 (PMC5878929; doi:10.7717/peerj.4573)
Supplement: Table S4 — Visual order of simulated movement treatments that correspond to the individual heat maps of each simulated disperser. The ‘heat map row’ corresponds to the visual row (1–27) on each heat map, while the number under Plot A, B and C (the plots representing results for Simulated Disperser A, B and C) specifies which treatment results are shown in the row. For example, heat map row 2 for simulated disperser A (A plot) corresponds to treatment 12 in Table 1. [file peerj-06-4573-s004.docx]

Supplementary table 4. Visual order of simulated movement treatments that correspond to the individual heat maps of each simulated disperser. The ‘heat map row’ corresponds to the visual row (1 – 27) on each heat map, while the number under Plot A, B and C (the plots representing results for Simulated Disperser A, B and C) specifies which treatment results are shown in the row. For example, heat map row 2 for simulated disperser A (A plot) corresponds to treatment 12 in Table 1.

| Heatmap row | A plot | B plot | C plot |
| --- | --- | --- | --- |
| 1 | 0 | 0 | 0 |
| 2 | 12 | 6 | 14 |
| 3 | 11 | 24 | 26 |
| 4 | 25 | 21 | 5 |
| 5 | 5 | 14 | 21 |
| 6 | 4 | 15 | 20 |
| 7 | 7 | 11 | 13 |
| 8 | 6 | 10 | 12 |
| 9 | 1 | 2 | 3 |
| 10 | 26 | 20 | 15 |
| 11 | 13 | 26 | 24 |
| 12 | 22 | 17 | 19 |
| 13 | 8 | 23 | 22 |
| 14 | 14 | 5 | 6 |
| 15 | 15 | 4 | 7 |
| 16 | 16 | 9 | 8 |
| 17 | 17 | 8 | 9 |
| 18 | 2 | 1 | 1 |
| 19 | 10 | 7 | 4 |
| 20 | 23 | 22 | 23 |
| 21 | 9 | 16 | 18 |
| 22 | 24 | 25 | 25 |
| 23 | 18 | 19 | 17 |
| 24 | 19 | 18 | 16 |
| 25 | 21 | 13 | 10 |
| 26 | 20 | 12 | 11 |
| 27 | 3 | 3 | 2 |

# Simulated Disperser Analysis: determining the number of loci required to genetically identify dispersers

Adam P.A. Cardilini^1^, Craig D.H. Sherman^2^, William B. Sherwin^3^, Lee A. Rollins^2^

^1^ Faculty of Science, Engineering and Built Environment, Deakin University, 221 Burwood Hwy, Burwood, Victoria 3125, Australia

^2^ Centre for Integrative Ecology, School of Life and Environmental Science, Deakin University, 75 Pigdons Rd, Waurn Ponds, Victoria 3216, Australia

^3^ School of Biological, Earth and Environmental Sciences, The University of New South Wales, High St, Kensington, NSW 2052, Australia

Keywords: Ecological Genetics, Population Genetics, Power Analysis, Migrant, GeneClass2

Corresponding Author:

Adam P.A. Cardilini

Faculty of Science, Engineering and Built Environment, Deakin University, 221 Burwood Hwy, Burwood, Victoria 3125, Australia

Email: a.cardilini@gmail.com
